# Early Endovenous Thermal Ablation With Concomitant Anticoagulation in Chronic Venous Insufficiency Complicated by Superficial Venous Thrombosis: A Retrospective Observational Study

**DOI:** 10.1016/j.ejvsvf.2026.03.006

**Published:** 2026-03-27

**Authors:** Jiasheng Zhang, Ruihao Li, Bingyao Zhang, Hui Yuan, Mao Zhang

**Affiliations:** a School of Medicine, University of Electronic Science and Technology of China, Chengdu, China; b Department of Vascular Surgery, Sichuan Provincial People's Hospital, University of Electronic Science and Technology of China, Chengdu, China; c Department of Clinical Medicine, Chongqing Medical University, Chongqing, China

**Keywords:** Ablation techniques, Anticoagulants, Chronic venous insufficiency, Superficial venous thrombosis, Varicose veins, Venous thrombosis

## Abstract

**Introduction:**

Whether endovenous thermal ablation (EVTA) can be performed soon after the diagnosis of superficial venous thrombosis (SVT) in patients with chronic venous insufficiency (CVI) remains uncertain. This study assessed the safety and clinical benefit of early EVTA performed under anticoagulation.

**Method:**

Consecutive CVI patients with acute SVT (symptom onset ≤14 days) who underwent endovenous laser ablation (EVLA), radiofrequency ablation (RFA), or endovenous microwave ablation (EMA) between January 2022 and March 2025 were identified. All received peri-procedural anticoagulation. Co-primary endpoints were technical success (first post-procedural duplex) and clinical improvement (change in Venous Clinical Severity Score [VCSS] to six months). Secondary endpoints included immediate pain relief (numerical rating scale [NRS] at rest and movement to discharge), Venous Insufficiency Epidemiological and Economic Study–Quality of Life (VEINES-QoL) change to six months, and complications (including deep vein thrombosis [DVT] and pulmonary embolism [PE]) and recurrence.

**Results:**

The study analysed 255 patients (262 limbs) with a median follow up of 14 months. Mean procedure duration was 54.3 ± 22.1 minutes. Complications were uncommon: DVT 1.2% (3 of 262), minor bleeding 0.78% (2 of 255); there was no PE, re-operation, or infection. Pain improved substantially (NRS decreased by 2.59 points at rest and 4.15 points with movement). Clinical severity and QoL also improved (VCSS decreased by 5.73 ± 1.78 points; VEINES-QoL increased by 12.87 ± 1.92 points). Outcomes and complication rates did not differ between EVLA, RFA, and EMA.

**Conclusion:**

In this cohort, early EVTA under anticoagulation appeared feasible and was associated with low thrombotic and bleeding complication rates and clinically meaningful improvements in pain, clinical severity, and quality of life. Given the non-controlled design, findings should be interpreted as hypothesis generating and warrant confirmation in comparative prospective studies.

## Highlights

• Retrospective cohort of 255 patients (262 limbs) with SVT and CVI.
• Early endovenous ablation with anticoagulation was feasible.
• Complications were rare: DVT 1.2%, with no pulmonary embolism.
• VCSS, VEINES-QOL, and pain improved significantly.

## INTRODUCTION

Chronic venous insufficiency (CVI) arises from valvular incompetence, venous obstruction, or previous thrombosis, leading to sustained venous hypertension and progressive anatomic and functional changes that impair quality of life. Primary (idiopathic) CVI accounts for approximately 70% of cases.^1^^,^^2^ Management of CVI is tailored to disease severity: mild cases are managed conservatively (compression therapy, exercise, limb elevation, etc.), while patients with significant symptoms and duplex confirmed reflux are candidates for interventional or surgical treatments.^3^, ^4^, ^5^, ^6^

CVI is an independent risk factor for superficial venous thrombosis (SVT), and the likelihood of SVT increases in more advanced CVI (Clinical–Etiologic–Anatomic–Pathophysiologic [CEAP] classification C4 – C6) compared with milder disease.^7^^,^^8^ In practice, patients with advanced CVI (especially C4 – C6) often require endovenous intervention to manage symptoms and prevent progression.^9^ However, when acute SVT coexists, the optimal timing of intervention remains uncertain. Expert opinion and the European Society for Vascular Surgery 2021 guidelines suggest deferring ablation after an SVT episode, but this is supported by low level evidence and may prolong symptoms.^10^ This recommendation is Level C evidence, reflecting weak data, and such delays may prolong patient discomfort and disease duration. Bikdeli *et al.* provided an up to date overview of SVT management, highlighting the limited evidence base for interventional approaches.^11^

To date, few studies have examined whether extended pre-operative anticoagulation is truly necessary in CVI patients with concurrent SVT. This study hypothesised that performing endovenous ablation early (soon after the SVT diagnosis) under anticoagulation coverage could be safe and beneficial. Therefore, it evaluated the safety and efficacy of early endovenous thermal ablation (EVTA) combined with post-operative anticoagulation in CVI patients with SVT who did not undergo prolonged pre-operative anticoagulation. The findings were aimed to inform peri-operative management and help bridge an important gap in current vascular practice.

## STUDY DESIGN

This single centre, retrospective cohort study was undertaken between January 2022 and March 2025. It identified all consecutive patients with lower extremity CVI (CEAP C2 – C6) who had undergone EVTA (endovenous laser ablation [EVLA], radiofrequency ablation [RFA], or endovenous microwave ablation [EMA]). A total of 255 patients (262 limbs) met the criteria and were analysed. This retrospective study was approved by the Ethics Committee of Sichuan Provincial People's Hospital (IRB No. 2025629; approval date: 9^th^ September 2025), and conducted in accordance with the Declaration of Helsinki. Individual consent was waived owing to use of de-identified data collected as part of routine care.

### For surgery

Intervention was indicated for: (1) CEAP C2 – C6 with clinically significant symptoms; (2) duplex confirmed axial reflux of the great saphenous vein (GSV) (reflux ≥0.5 seconds) with saphenous valve incompetence; (3) failure of conservative management (compression, elevation, venoactive drugs); and (4) a patent deep venous system, with no acute or chronic deep vein thrombosis (DVT) on ultrasound.

### Patient selection

Inclusion criteria required patients to have an acute presentation of lower extremity superficial thrombosis (painful superficial vein inflammation) with symptom onset ≤14 days before admission, and duplex ultrasound confirmation of thrombosis in the GSV, with axial reflux (≥0.5 seconds) documented in the patent portion of the same refluxing saphenous trunk (adjacent to or proximal to the thrombus). Reflux was only assessed in patent venous segments and was not measured across a fully thrombosed occluded segment.

Exclusion criteria were: SVT located within 15 cm of the saphenofemoral junction (to ensure an adequate working length for proximal ablation); thrombosis extending through the entire length of the superficial venous system in the affected limb; a previous history of DVT; known chronic (non-acute) SVT; lack of anticoagulant therapy during treatment; or incomplete follow up data.

### Endovenous ablation therapy

**Pre-operative preparation**. On admission, baseline numerical rating scale (NRS) (rest and movement), VCSS (Venous Clinical Severity Score), and VEINES-QoL (Venous Insufficiency Epidemiological and Economic Study–Quality of Life) were recorded. Detailed venous mapping documented varicosities, tributaries, and SVT location. Incompetent perforator veins were assessed on baseline duplex ultrasound. All patients received subcutaneous enoxaparin 1 mg/kg once daily before the procedure; the last dose was given ∼12–24 hours before surgery.

**Operative steps**. Procedures were performed with the patient under general anaesthesia in the supine position. General anaesthesia was selected because the index intervention frequently combined multiple steps (e.g., dual site access in some cases, adjunctive foam sclerotherapy, micro-incisional thrombus extraction, and short segment phlebectomy or stripping), for which the patient's comfort and immobility were required. Tumescent infiltration (normal saline infiltration) was infiltrated around the target vein segment. Local anaesthetic was omitted because procedures were performed under general anaesthesia. Endovenous instruments included a radiofrequency closure catheter CF7-7-60 (Medtronic, Minneapolis, MN, USA), microwave ablation catheter ECO-100F-2016 (Nanjing ECO Microwave System Co., Ltd., Nanjing, China), and endovenous laser ablation optical fibre CS-B 400×2.5 (Chunhui Technology, China). The treatment approach was tailored according to the thrombus location: for planning purposes, cases were categorised as either thigh segment thrombosis or calf segment thrombosis. In both scenarios, EVTA was combined with adjunctive therapies, specifically: foam sclerotherapy to obliterate varicose tributaries, micro-incisional thrombus extraction (via a small stab incision over the thrombosed segment), and short segment saphenous trunk stripping. Perforator directed treatment was not routinely performed during the index procedure.

**Thigh segment thrombosis.** The following interventions were performed for thigh segment thrombus (GSV, thigh).

***Two stage EVTA with adjunctive interventions*.** When thrombus was confined to the thigh segment of the GSV, a two site strategy was used to thermally isolate the clot, followed by adjunctive interventions.

Stage 1: Proximal segment ablation. Under duplex guidance, the GSV was cannulated percutaneously ∼1 cm cranial to the thrombus and intraluminal position confirmed by free venous backflow. A 6 Fr introducer sheath was inserted. The RFA probe, 810 nm EVLA fibre, or EMA antenna was advanced and positioned ∼2 cm distal to the saphenofemoral junction (SFJ). After circumferential tumescent infiltration, segmental thermal ablation was performed with controlled pullback to the cranial margin of the thrombus using device specific parameters (RFA ∼120 °C; 810 nm EVLA 12 W; EMA 55 W in five second pulses), thereby sealing the proximal outflow and isolating the clot.
Stage 2: Distal segment ablation via routine access. Via routine knee level GSV access, the catheter was advanced to ∼ one cm caudal to the thrombus; segmental ablation completed distal inflow occlusion using the same parameters.

***Adjunctive interventions*.** After EVTA, adjuncts were standardised: tributaries >5 mm underwent short segment phlebectomy or stripping; 1–3 mm veins received ultrasound guided segmental foam sclerotherapy (1% polidocanol, liquid to air 1:4, total ≈10 mL); <1 mm veins received focal injections. A ∼5 mm stab incision over the thrombosed segment enabled gentle micro-incisional thrombus extraction, with excision confined to the clot bearing short segment, to reduce the local thrombotic and inflammatory burden.

**Calf segment thrombosis.** The following interventions were performed for thigh segment thrombus (GSV, calf).

***Single access EVTA*.** For thrombus confined to the calf segment of the GSV, a single stage procedure was used. Under duplex guidance, the GSV was punctured just at the knee level via the routine knee level GSV access, a sheath was inserted, and the catheter tip was advanced to ∼2 cm distal to the SFJ. After longitudinal tumescent infiltration along the thigh segment, a continuous segmental ablation was performed in one pullback from proximal to distal, using the same device parameters.

***Adjunctive management and thrombus removal*.** As indicated, ultrasound guided foam sclerotherapy with 1% polidocanol (1:4 liquid to air ratio, ∼10 mL total) was used to treat residual tributaries; larger branches were addressed by micro-incisional stripping. Micro-incisional thrombus extraction was performed through a ∼5 mm incision over the thrombus, followed by meticulous haemostasis and compression dressing. All limbs were subsequently wrapped to maintain therapeutic compression.

**Post-operative management**. In this retrospective cohort, baseline mobility limitation was not systematically captured as a structured variable; however, most patients were independently ambulatory at presentation, with a minority requiring walking aids. No routine pre-operative bed rest was prescribed. Post-operatively, early ambulation was encouraged. Each treated limb was covered with a sterile dressing and an elastic compression bandage for 48 hours. Thereafter, patients were prescribed 20–30 mmHg graduated compression stockings for at least four weeks (continuous wear for the first two weeks, followed by daytime wear for a further two weeks). This extended regimen was adopted because many patients had advanced CVI with acute SVT associated inflammation, and some underwent adjunctive micro-incisions; in this context, compression was intended to limit bruising and haematoma, reduce inflammatory pain, and support skin and wound care. The regimen was modified as needed for cases of intolerance or skin related adverse effects. Patients with venous ulcers received regular wound care with sterile gauze changes every three days. At discharge, all patients were started on a one month course of a direct oral anticoagulant (either apixaban 2.5 mg twice daily or rivaroxaban 10 mg once daily). Doses were adjusted as necessary for renal and hepatic function. Anticoagulation was stopped immediately if any bleeding complication occurred.

### Follow up and assessments

The index date was the procedure date. Baseline VCSS, VEINES-QoL, and NRS (rest/movement) were recorded; NRS was reassessed at discharge (24–48 h). Outpatient visits were scheduled at one, three, and six months with standardised clinical examination and duplex by credentialed sonographers. Perforators were reassessed during follow up and treated only if clinically indicated. The frequency and type of any additional perforator targeted interventions during follow up were not systematically recorded and analysed in this study. Technical success was assessed at the first post-procedure duplex. VEINES-QoL and VCSS were repeated at six months.

### Outcome measures and definitions

Unless otherwise stated, procedural and thrombotic events were reported per treated limb, whereas symptom and QoL scores are reported per patient. Bleeding events were reported per patient.

Co-primary outcomes were: (1) Technical success: complete occlusion of the treated saphenous trunk (GSV and accessory GSV) with no colour Doppler flow on the first post-procedural duplex;^3^ (2) Clinical improvement: reduction in VCSS from baseline to six months, calculated as baseline VCSS minus six month VCSS (positive values indicate improvement).

Secondary outcomes were: (1) Immediate pain relief: reduction in NRS at rest and with movement from baseline to discharge, calculated as baseline NRS minus discharge NRS (positive values indicate improvement); (2) Quality of life: change in VEINES-QoL from baseline to six months, calculated as six month minus baseline (positive values indicate improvement); (3) Treatment failure: incomplete ablation of the target vein (e.g., >10 cm of persistent patency or any residual segment with significant reflux on imaging); (4) Recurrence (time to event): reappearance or persistence of venous disease after surgery, defined as (i) new varicosities or recurrent symptoms and or (ii) imaging confirmed recanalisation or neovascularisation of the previously treated trunk or tributaries at follow up.^12^ VCSS (revised 2010), VEINES-QoL (T score method), and 0–10 NRS were applied (Supplementary Material S1).

### Complications

Adverse events were graded using the Society of Interventional Radiology specialty specific classification.^13^^,^^14^

### Statistical analysis

Continuous variables were tested for normality (Shapiro–Wilk). Normally distributed data (mean ± standard deviation) were compared across EVLA, RFA, and EMA using one way ANOVA with Tukey/LSD *post hoc* tests; non-normal data (median [interquartile range, IQR]) used Kruskal–Wallis with Dunn *post hoc*. Categorical variables (*n*, %) used chi square or Fisher's exact test when expected counts <5. All tests were two sided; *p* < .05 was considered statistically significant. R version 4.4.2 (R Foundation for Statistical Computing, Vienna, Austria).

## RESULTS

**Figure 1:**
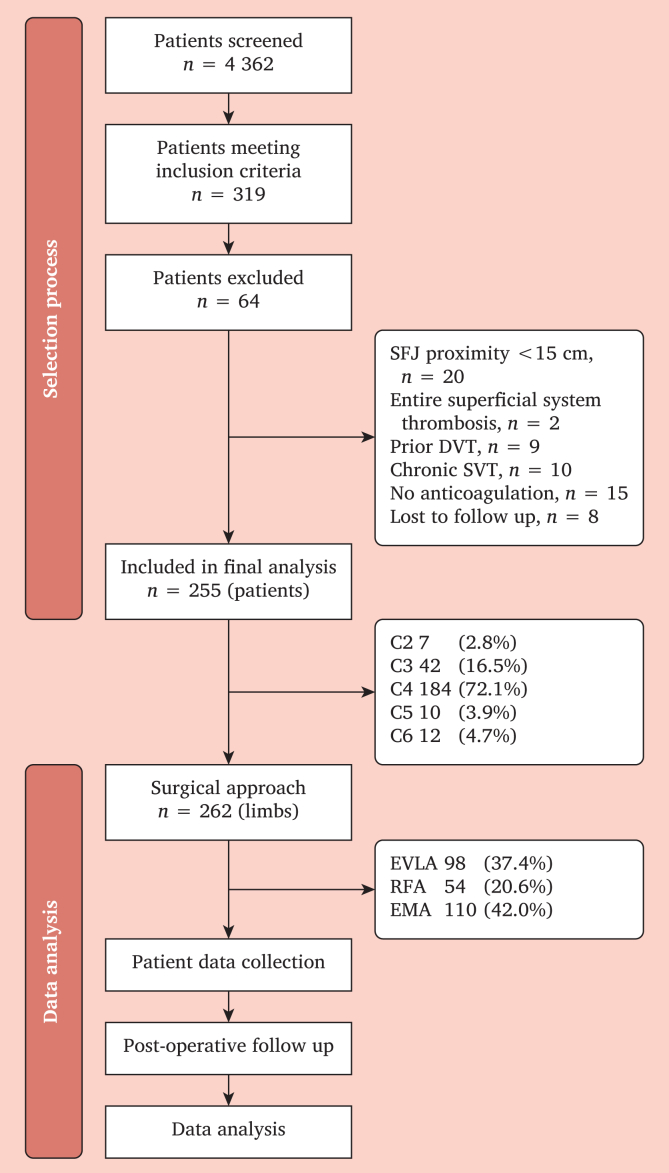
Flowchart of patient screening, inclusion, and exclusion throughout the study (4 362 screened; 319 met entry criteria; 255 patients [262 limbs] analysed).

As shown in Fig. 1, 4 362 patients were screened; 319 met entry criteria. After exclusions (SFJ proximity <15 cm, *n* = 20; entire superficial system thrombosis, *n* = 2; previous DVT, *n* = 9; chronic SVT, *n* = 10; no anticoagulation, *n* = 15; lost to follow up, *n* = 8), 255 patients (262 limbs) were analysed.

Baseline characteristics are shown in Table 1. The cohort comprised 255 patients with a mean age of 61.2 ± 11.6 years, and 28 (11.0%) were aged ≥75 years; sex distribution was balanced (50.2% women). Mean body mass index (BMI) was 24.8 ± 3.3 kg/m^2^, with obesity (BMI ≥30 kg/m^2^) in eight patients (3.1%). Smoking and regular alcohol use were reported in 23.5% and 25.5%, respectively. Hypertension (16.1%) and diabetes mellitus (9.0%) were the most common comorbidities, and median follow up was 14 months (IQR 6, 30).

**Table 1:** Patient baseline demographic and clinical characteristics (patient level summary *n* = 255).

Variable	Total
Patients – *n*	255
Age – y	61.2 ± 11.6
Age ≥75 y	28 (11.0)
BMI – kg/m^2^	24.8 ± 3.3
BMI ≥30 kg/m^2^	8 (3.1)
Follow up – mo	14 (6, 30)
Men	127 (49.8)
Women	128 (50.2)
Smoking	60 (23.5)
Alcohol use	65 (25.5)
Hypertension	41 (16.1)
Diabetes	23 (9.0)
Coronary artery disease	1 (0.4)

Data are presented as *n* (%), mean ± standard deviation, or median (interquartile range), as appropriate. BMI = body mass index.

Supplementary Table S1 summarises venous disease characteristics. In this cohort, 27.8% of patients had bilateral CVI, 27.8% had disease confined to the right leg, and 44.3% had disease confined to the left leg. By CEAP clinical class (patient level), 2.8% were C2, 16.5% C3, 72.2% C4, 3.9% C5, and 4.7% C6.

Regarding the location of the SVT: 56.5% of patients (144 of 255) had left leg SVT and 40.8% (104 of 255) had right leg SVT, while 2.7% had bilateral SVT. Most thromboses were in the distal (below knee) portion of the saphenous system. Specifically, of the SVTs in left legs, 10 were in the proximal thigh and 134 in the distal calf; on the right side, eight were proximal and 96 distal. All bilateral cases involved only below knee thromboses.

Of 262 treated limbs, 98 (96 patients) underwent EVLA, 54 (54 patients) RFA, and 110 (105 patients) EMA. The median pre-operative D dimer level was 0.75 μg/mL (IQR 0.32, 1.36), and the mean procedure time was 54.3 ± 22.1 minutes (Supplementary Table S2).

Post-operative outcomes are shown in Table 2. Varicose vein recurrence occurred in four of 262 (1.53%) limbs. There were no re-operations, pulmonary emboli (PE), or post-operative infections. Three limbs (1.15%) developed a new DVT post-operatively, and minor post-operative bleeding occurred in two of 255 (0.78%) patients. In the baseline CEAP C6 subgroup (*n* = 12), ulcer healing at six months was complete in six (50.0%), partial in four (33.3%), and unhealed in two (16.7%) patients.

**Table 2:** Post-operative outcomes and changes in symptom and quality of life scores∗.

Variable	Total
Recurrence	4 (1.53)
Re-operation	–
Pulmonary embolism	–
Post-operative Infection	–
Deep vein thrombosis	3 (1.15)
Post-operative bleeding	2 (0.78)
VCSS score improvement‡	5.73 ± 1.78 (5.51–5.95)
VEINES-QoL score improvement‡	12.87 ± 1.92 (12.63–13.11)
NRS pain score improvement (Rest)†	2.59 ± 1.08 (2.46–2.72)
NRS pain score improvement (Movement)†	4.15 ± 1.28 (3.99–4.31)
Ulcer complete healing at six months (C6 only)	6/12 (50.0)
Ulcer partial healing at six months (C6 only)	4/12 (33.3)
Ulcer unhealed at six months (C6 only)	2/12 (16.7)

Data are presented as *n* (%) or mean ± standard deviation, with 95% CIs for continuous outcomes (*t* distribution). NRS = numerical rating scale; VCSS = Venous Clinical Severity Score; VEINES-QoL = Venous Insufficiency Epidemiological and Economic Study–Quality of Life; – = not observed.

∗ Recurrence, re-operation, pulmonary embolism, post-operative infection, and deep vein thrombosis are limb level outcomes (*n* = 262). Post-operative bleeding is a patient level outcome (*n* = 255). Ulcer status at six months is reported for the baseline CEAP C6 subgroup (patient level; *n* = 12).

† Assessed at discharge.

‡ Assessed at six months.

Symptom severity and quality of life markedly improved after treatment. On average, the NRS pain score decreased by 2.59 ± 1.08 points at rest and by 4.15 ± 1.28 points during movement. The VCSS decreased by 5.73 ± 1.78 points. Similarly, the VEINES-QoL score increased by 12.87 ± 1.92 points.

Outcomes were compared across EVLA, RFA, and EMA; no between group differences were detected for pain relief, VCSS, VEINES-QoL, or complication rates.

**Figure 2:**
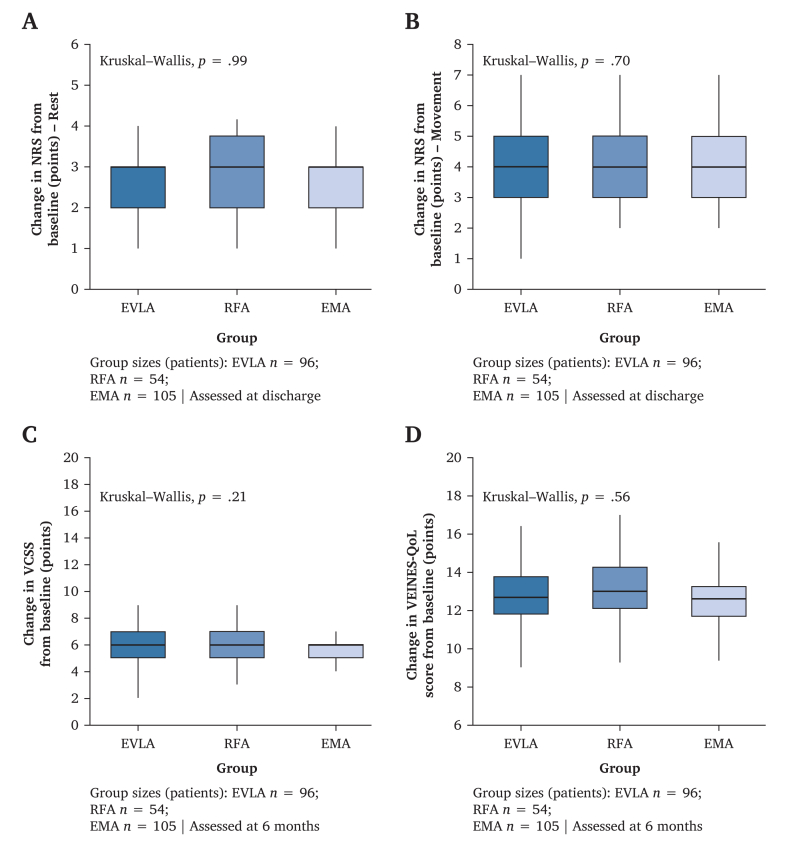
Clinical outcomes by treatment group after early endovenous thermal ablation under anticoagulation. (A) Change in numerical rating scale (NRS) pain at rest from baseline to discharge (∼24–48 h); median improvement ≈2.5 points across groups (Kruskal–Wallis, *p* = 0.99). (B) Change in NRS pain during movement to discharge; median improvement ≈4.0 points (*p* = 0.70). (C) Change in Venous Clinical Severity Score (VCSS) from baseline to six months; median improvement ≈6 points (*p* = 0.21). (D) Change in VEINES-QoL from baseline to six months; median improvement ≈13 points (*p* = 0.56). No between group differences were detected in any panel. Changes are oriented so that positive values indicate improvement (baseline minus follow up for NRS and VCSS; follow up minus baseline for VEINES-QoL). EVLA = endovenous laser ablation; RFA = radiofrequency ablation; EMA = endovenous microwave ablation; VEINES-QoL = Venous Insufficiency Epidemiological and Economic Study–Quality of Life.

The median NRS improvements were ≈2.5 (rest) and 4.0 (movement) in each group (Kruskal–Wallis: rest *p* = 0.99; movement *p* = 0.70). The median VCSS improvements were ≈6 in all groups (*p* = 0.21). VEINES-QoL improved ≈13 points, without intergroup differences (*p* = 0.56) (Fig. 2).

Table 3 compares recurrence and complication rates across the EVLA, RFA, and EMA groups. There were no significant differences between groups in any complication or recurrence outcome. Recurrence rates were uniformly low (*p* = 0.79). Post-op DVT: 0% (EVLA), 3.7% (RFA), and 0.9% (EMA). Minor bleeding occurred only in EMA (1.9%). No PE, re-operations, or post-operative infection occurred in any group.

**Table 3:** Post-operative recurrence and complication rates by ablation modality∗.

Complication	EVLA (*n* = 98)	RFA (*n* = 54)	EMA (*n* = 110)	*p* value (overall)
Recurrence	2 (2.0)	1 (1.9)	1 (0.9)	0.8
Re-operation	0 (0)	0 (0)	0 (0)	–
Pulmonary embolism	0 (0)	0 (0)	0 (0)	–
Post-operative infection	0 (0)	0 (0)	0 (0)	–
Deep vein thrombosis	0 (0)	2 (3.7)	1 (0.9)	0.1
Post-operative bleeding†	0 (0)	0 (0)	2 (1.9)	0.2

Data are presented as *n* (%). EVLA = endovenous laser ablation; RFA = radiofrequency ablation; EMA = endovenous microwave ablation; – = not observed.

∗ Limb level outcomes by ablation modality unless otherwise specified.

† Post-operative bleeding is reported per patient (EVLA *n* = 96; RFA *n* = 54; EMA *n* = 105). Overall (3×2) comparisons used Pearson's χ^2^; given small, expected counts, Fisher–Freeman–Halton exact (or χ^2^ with Monte-Carlo p) may be used for confirmation.

## DISCUSSION

Most SVTs occur in patients with varicose veins,^15^ yet evidence based guidance for CVI complicated by acute SVT remains limited. In this single centre, retrospective cohort (*n* = 255), early EVTA performed under anticoagulation was feasible and associated with clinically relevant improvements in pain, VCSS, and VEINES-QoL.

The mean procedure duration (∼54 minutes) was slightly longer than that reported in standard endovenous ablation series,^16^^,^^17^ plausibly reflecting the additional steps required in the acute SVT setting (e.g., mapping and management of the clot bearing superficial segment). Recurrence was low (∼1.5%) at a median follow up of 14 months, and was comparable with published outcomes after endovenous ablation.^16^^,^^18^ Rapid analgesic benefit was observed, consistent with previous reports of early pain reduction after ablation.^19^ Improvements in VCSS and VEINES-QoL were also within the ranges typically reported after endovenous ablation,^20^^,^^21^ including in patients with more advanced CVI.^20^, ^21^, ^22^, ^23^

Safety signals were reassuring. Post-operative DVT occurred in ∼1.15% of treated limbs; while numerically higher than pooled rates after routine EVTA (∼0.2–0.5%);^24^ this may reflect the increased baseline thrombotic risk in patients presenting with SVT, and the absolute rate remained low and comparable with other EVTA series.^16^ Bleeding events were infrequent (two minor, self limited epistaxis episodes), in keeping with reports showing very low rates of clinically relevant bleeding after CVI interventions.^25^ No PE, deep infection, or re-intervention occurred, consistent with the low rates reported after EVTA,^20^^,^^24^ and early re-operation for recurrent varicose veins was generally uncommon in the first one to two years.^18^

This study had some limitations. The retrospective, single cohort design without a contemporaneous control group and the low event rates limited causal inference and the precision of estimates for rare outcomes. Early EVTA was not directly compared with alternative interventional strategies such as catheter based aspiration or mechanical thrombectomy; the role of these techniques in SVT remains undefined and warrants prospective comparative evaluation. Moreover, the incremental contribution of clot segment micro-incisional management could not be determined within this non-comparative design. Established MCIDs for VCSS and VEINES-QoL in this setting are lacking; therefore, absolute score changes with confidence intervals are reported rather than applying unvalidated thresholds. Finally, follow up was limited to a median of 14 months, and longer term durability beyond one year should be confirmed in prospective, multicentre studies.

### Conclusion

In this retrospective cohort of patients with chronic venous insufficiency presenting with acute superficial venous thrombosis, early truncal endovenous thermal ablation performed under anticoagulation coverage was feasible and associated with low rates of thrombotic or bleeding complications, alongside meaningful improvements in pain, VCSS, and VEINES-QoL. Prospective comparative studies are warranted to define optimal timing, patient selection, and adjunctive strategies.

## Funding

No specific funding.

## DATA AVAILABILITY

De-identified data and analysis code are available upon reasonable request from the corresponding author; sharing is subject to institutional policies and patient privacy regulations.

## USE OF GENERATIVE AI TOOLS

During the preparation of this work, the authors used ChatGPT (OpenAI, San Francisco, CA, USA) to improve English language and readability. The authors reviewed and edited the content as needed and take full responsibility for the content of the publication.

## CONFLICT OF INTEREST

The authors have no competing interests.
