# Supplementary material for: Early Endovenous Thermal Ablation With Concomitant Anticoagulation in Chronic Venous Insufficiency Complicated by Superficial Venous Thrombosis: A Retrospective Observational Study
Source: EJVES Vasc Forum. 2026 Mar 27;65:187–94. doi: 10.1016/j.ejvsvf.2026.03.006 (PMC13138230; doi:10.1016/j.ejvsvf.2026.03.006)
Supplement: Multimedia component 3 [file mmc3.docx]

**Table S2** Operative details of endovenous treatments(limb-level)^[[1]](#footnote-1)^

| **Variable** | **Total** |
| --- | --- |
| Number of Procedures (limb-level)  Technical success at first post-procedure duplex, % (95% CI)  D-dimer (µg/mL FEU)  Operation Type | 262 limbs  100% (98.6%-100%)  0.75 (0.32–1.36) |
| EVLA | 98 (37.4%) |
| RFA | 54 (20.6%) |
| EMA | 110 (42.0%) |
| Duration of Surgery, min | 54.3±22.1 |

1. Notes: Limb-level summary (N = 262). Technical success assessed at the first post-procedure duplex (≈1-month visit); 95% CI by exact (Clopper–Pearson) method. D-dimer is reported as median (IQR). Duration refers to skin-to-skin time. Abbreviations: EVLA, endovenous laser ablation; RFA, radiofrequency ablation; EMA, endovenous microwave ablation; FEU, fibrinogen-equivalent units. [↑](#footnote-ref-1)
