# Supplementary material for: Early Endovenous Thermal Ablation With Concomitant Anticoagulation in Chronic Venous Insufficiency Complicated by Superficial Venous Thrombosis: A Retrospective Observational Study
Source: EJVES Vasc Forum. 2026 Mar 27;65:187–94. doi: 10.1016/j.ejvsvf.2026.03.006 (PMC13138230; doi:10.1016/j.ejvsvf.2026.03.006)
Supplement: Multimedia component 4 [file mmc4.docx]

**Supplementary Table S1. Venous disease distribution and SVT location (patient level).^[[1]](#footnote-1)^**

| **Variable** | **Total** |
| --- | --- |
| Laterality of CVI |  |
| Left | 113 (44.3%) |
| Right | 71 (27.8%) |
| Bilateral | 71 (27.8%) |
| CEAP Classification |  |
| C2 | 7 (2.8%) |
| C3 | 42 (16.5%) |
| C4 | 184 (72.2%) |
| C5 | 10 (3.9%) |
| C6 | 12 (4.7%) |
| SVT location |  |
| Left thigh | 10 (3.9%) |
| Left calf | 134 (52.6%) |
| Right thigh | 8 (3.1%) |
| Right calf | 96 (37.7%) |
| Bilateral calf | 7 (2.7%) |

1. Notes: Patient-level summary (N = 255). Thigh and calf denote proximal and distal segments, respectively, as defined by duplex mapping; acute SVT onset ≤14 days. Data are n (%).

   Abbreviations: CEAP, Clinical–Etiology–Anatomy–Pathophysiology; SVT, superficial venous thrombosis. [↑](#footnote-ref-1)
