# Supplementary material for: Early Endovenous Thermal Ablation With Concomitant Anticoagulation in Chronic Venous Insufficiency Complicated by Superficial Venous Thrombosis: A Retrospective Observational Study
Source: EJVES Vasc Forum. 2026 Mar 27;65:187–94. doi: 10.1016/j.ejvsvf.2026.03.006 (PMC13138230; doi:10.1016/j.ejvsvf.2026.03.006)
Supplement: Multimedia component 6 [file mmc6.docx]

Supplementary Material S1. Scoring Sheets.

This supplementary file summarizes the structure, scoring rules, and interpretation for three widely used outcome measures in chronic venous disease and peri‑procedural pain assessment: the Venous Clinical Severity Score (VCSS, revised 2010), the VEINES‑QoL/Sym questionnaire, and the 0–10 Numeric Rating Scale (NRS) for pain.

# 1) Venous Clinical Severity Score (VCSS, revised 2010)

Purpose: clinician‑reported index to quantify disease severity and change over time in chronic venous disease. The revised VCSS clarifies each descriptor and uses four grades (0 = none, 1 = mild, 2 = moderate, 3 = severe). Ten descriptors are summed to a total score ranging from 0 to 30; higher scores indicate more severe disease. ^1^

## Descriptors and grading (0–3)

| Descriptor | 0 — None | 1 — Mild | 2 — Moderate | 3 — Severe |
| --- | --- | --- | --- | --- |
| Pain / discomfort (presumed venous origin) | No pain or discomfort | Occasional; does not restrict routine activities | Daily; interferes with but does not prevent routine activities | Daily; limits most routine activities |
| Varicose veins (≥3 mm when standing) | Absent | Few, scattered clusters; may include corona phlebectatica | Confined to calf or thigh | Involves both calf and thigh |
| Venous edema (presumed venous origin) | Absent | Limited to foot/ankle region | Extends above ankle but below knee | Extends to knee and above |
| Skin pigmentation (venous origin; exclude focal pigmentation over veins or other diseases) | None or focal only | Limited to perimalleolar area | Diffuse over lower third of calf | Wider distribution above lower third of calf |
| Inflammation (e.g., erythema, cellulitis, venous eczema/dermatitis) | Absent | Limited to perimalleolar area | Diffuse over lower third of calf | Wider distribution above lower third of calf |
| Induration / lipodermatosclerosis / atrophie blanche (venous origin) | Absent | Limited to perimalleolar area | Diffuse over lower third of calf | Wider distribution above lower third of calf |
| Number of active ulcers | 0 | 1 | 2 | ≥3 |
| Duration of active ulcer (longest) | N/A (no active ulcer) | < 3 months | > 3 months and < 1 year | Not healed for ≥ 1 year |
| Size of active ulcer (largest) | N/A (no active ulcer) | ≤ 2 cm diameter | 2–6 cm diameter | ≥ 6 cm diameter |
| Compression therapy use | Not used | Intermittent use of stockings | Wears stockings most days | Full compliance with stockings |

*Administration: one limb per line; if both limbs are assessed, score separately. Sum the ten descriptors for the limb total (0–30).*

# 2) VEINES‑QoL/Sym Questionnaire

Purpose: patient‑reported measure for symptoms and health‑related quality of life in chronic venous disorders of the leg (CVDL). The instrument contains 26 items; 25 items contribute to the VEINES‑QoL summary score (higher scores = better status), and a 10‑item subset forms the VEINES‑Sym symptom score. ^2,3,4^

## Structure

Items cover symptoms (10 items), daily activity limitation (9), psychological impact (5), one item on change over 1 year, and one item on time‑of‑day when symptoms are worst. Question 2 (time‑of‑day) is descriptive and not included in scoring for VEINES‑QoL. VEINES‑Sym uses items 1a–1i and 7.

## Scoring and missing data

Code each response numerically so that higher values reflect better health/status per the instrument’s coding scheme. A common scoring approach is the cohort‑relative T‑score method: standardize each scored item within the sample to a z‑score, average across items, then linearly transform to T‑scores (mean 50, SD 10). This yields scores comparable within a study cohort.

An alternative “intrinsic” 0–1 rescaling (average of rescaled items) facilitates between‑study comparisons in venous leg ulcer research and is recommended by some validation work. ^4^

Handling missing items: compute VEINES‑QoL if ≥50% of items are present and VEINES‑Sym if ≥5 of the 10 symptom items are present; otherwise set the scale to missing.

*Directionality: higher VEINES‑QoL and VEINES‑Sym indicate better quality of life / fewer symptoms. Report the specific scoring method used (relative T‑score vs intrinsic 0–1 rescaling) in the Methods.*

# 3) Numeric Rating Scale (NRS) for Pain (0–10)

Purpose: patient‑reported measure of pain intensity used at rest and during movement. Participants select an integer from 0 to 10 that best represents their pain intensity over a specified recall period (commonly “now” or “last 24 hours”). ^5,6^

## Anchors and administration

Scale anchors: 0 = no pain; 10 = worst pain imaginable. Administration can be verbal or written; typical completion time is < 3 minutes; minimal training required.

# References

1. Vasquez MA, Rabe E, McLafferty RB, Shortell CK, Marston WA, Gillespie D, et al. Revision of the venous clinical severity score: venous outcomes consensus statement of the American Venous Forum. J Vasc Surg. 2010;52(5):1387–1396.

2. Lamping DL, Schroter S, Kurz X, Kahn SR, Abenhaim L. Evaluation of outcomes in chronic venous disorders of the leg: development of a scientifically rigorous, patient‑reported measure of symptoms and quality of life. J Vasc Surg. 2003;37(2):410–419.

3. Kahn SR, Lamping DL, Ducruet T, Arsenault L, Miron MJ, Roussin A, et al. VEINES‑QoL/Sym questionnaire was a reliable and valid disease‑specific quality of life measure for deep venous thrombosis. J Clin Epidemiol. 2006;59(10):1049–1056.

4. Bland JM, Dumville JC, Ashby RL, Gabe R, Stubbs N, Adderley U, et al. Validation of the VEINES‑QoL quality of life instrument in venous leg ulcers: repeatability and validity study embedded in a randomised clinical trial. BMC Cardiovasc Disord. 2015;15:85.

5. Shirley Ryan AbilityLab. Numeric Pain Rating Scale. Rehabilitation Measures Database. Available at: https://www.sralab.org/rehabilitation-measures/numeric-pain-rating-scale. Accessed 27 Sep 2025.

6. Olsen MF, Bjerre E, Hansen MD, Hilden J, Landler NE, Tendal B, et al. Pain relief that matters to patients: systematic review of empirical studies assessing the minimum clinically important difference in acute pain. BMC Med. 2017;15:35.
